# The Navy and Our Sailors

**Published:** 1918-03-23

**Authors:** 


### THE NAVY AND OUR SAILORS.

### A Memorable Annual Meeting.

THE Dreadnought Seamen's Hospital has had many famous annual meetings and dinners, but the meeting held on the 14th instant at the Prince's Restaurant, at which the First Sea Lord presided, promises to be memorable for all time. It must have been full of inspiration for Sir Rosslyn Wemyss, who rendered excellent personal service in consenting to preside, and enabling the public and the sailors to show by their presence the esteem, welcome, and confidence extended to the Navy and himself throughout the nation. Everyone who attended was glad to be there, because the speeches were redolent of the sea, as this report demonstrates.

**Sir Perceval Nairne,** the Chairman, read the annual report, which stated that the patients included men who had fought in almost every naval engagement since the war began, men of the merchant service, mine-layers, mine-sweepers, and patrol boat men. There were several instances of men going to sea again and again after serving in torpedoed ships. As an instance of the sufferings of sailors, the report mentioned the case of the steamer *Sagamore*, torpedoed in the North Sea in the early part of the year, when of seventeen men abandoned in an open boat twelve died. The others suffered so grievously from frost-bite that they all had to have their lower limbs removed. With the aid of the British Red Cross Society and the Order of St. John they were fitted with artificial limbs at the Seamen's Hospital, and were now able to walk. No progress had been made in improving the hospital buildings at Greenwich, but careful consideration was being given to the scheme for improvement and endowment, now practicable through the splendid fund Lord Devonport had collected.

### THE FIRST SEA LORD'S SPEECH.

**Sir Rosslyn Wemyss, K.C.B.** (*The Times* report), said: He was delighted to testify by his presence to the regard of the Admiralty for the merchant service, their comrades in arms. The object of the gathering appealed to the instinct of every Englishman. Before the war they were perpetually told that this country depended upon sea-borne traffic for food and raw materials for industry, and the war had given a practical lesson in that direction.

The enemy had attacked our merchant marine, but had not found the weakness he had expected; he did not realise that dogged perseverance in duty and that persistency of effort for which our race had ever been conspicuous. Our merchant navy had proved itself to be, as it always was, manned by men whom fear did not deter from duty and who bore their share in the war in a manner that upheld the best traditions of the sea. It behoved all who were interested in their efforts to see that these men were properly looked after. The action of the enemy had taught us that the Royal Navy and the mercantile marine depended one on the other. No longer could it be said that the merchant seaman could follow his trade in comparative safety when the seas were swept, as now, of the enemy's cruisers. He had now to combat a menace more threatening, more ruthless, more barbarous and treacherous than the piracies of the Middle Ages. It was a duty to make the future of the merchant seaman assured, and in a practical manner this could be done by helping the hospital and its staff at Greenwich. If the appeal met with the success it deserved, there was much to be done for care of pulmonary complaints and for the comfort of the incurable.

As to the site of the hospital, the Admiralty were most anxious to do all in their power to further any scheme for the benefit of the hospital and were willing to meet the committee of management in their views. If it was decided that the present site was the best, the Admiralty would grant a new lease without the present resumption clause; in any circumstances the Admiralty as trustees would require that plans and architecture should harmonise with the whole of the Greenwich Hospital building. Should the committee decide upon some other site the Admiralty would assist the new undertaking and would be prepared to resume possession of the present building. He felt that a fine building at Greenwich would be a worthy memorial to the gallantry of men of the merchant service.

**Sir Maurice de Bunsen** said in his profession during forty years he had seen in remote regions what was the meaning of the British Empire, how poor the world would be without it, and what a mighty instrument it was for good. It had been brought home to him many a time, and there was no more cheering sight to a diplomatist at his post than to see the British Flag, whether it were the red ensign, the blue ensign, or the white ensign, coming into some foreign port, and showing the intimate connection which is kept up by our great merchant and naval services between the remotest lands and the Home country. It was owing to their services that the British Empire was intact; it stood to-day in absolute unanimity, and it had been closely welded as we had proceeded through the war, a fact which should bring pride and confidence to everybody when the world was cracking and splitting up over the greater part of it. These facts cheered him as he joined his testimony to that of the gallant Admiral as to the indescribable service rendered from day to day by these great services. Further, it was welcome to hear of the new buildings that were in prospect to increase the size of the Dreadnought. No man or woman could doubt that the members of the Mercantile Marine, when they came home to their own country, should find every accommodation ready for their reception and care.

### SPLENDID FIGURES.

**Lord Inchcape** testified to the yeoman service done for sailors, than whom there is no more deserving body in the land. From small beginnings in 1821 the Seamen's Hospital had passed through its portals no fewer than 934,000 men, and in 1917 alone 17,000 men were treated in the hospital. He welcomed the great interest which the Admiralty felt in this great undertaking, the Dreadnought Hospital. So far as the Navy was concerned, and the captains, officers, engineers, seamen, and firemen of the Merchant Service, no slackers, conscientious objectors, and persons of that kidney, could be found. Men whose ships had been torpedoed or mined, not once, but several times, to Lord Inchcape's knowledge, and, in the case of many of his own ships, many men who had undergone great suffering, had turned up again smiling, asking for another ship. We were a seafaring race, and a seafaring race until the end of time we should continue to be. The whole world was familiar to us. We felt as much at home on the coast of India, of China, the Persian Gulf, Australasia, the Continent of America, Scandinavia, the Red Sea, the Mediterranean, the White Sea, and the Black Sea as we did in navigating round the British

### THE NAVY AND OUR SAILORS—(*continued*).

Islands. We had, heretofore, felt equally at home in the Baltic and the Black Sea, and please God, we should before long feel at home in these waters again. He hoped Sir Rosslyn Wemyss would have his eyes in the direction of the Gulf of Heligoland, towards which, as a man of Fife, he knew the "East Nuik" is poking its nose.

THE ANNUAL MEETING'S SPLENDID HELP.

**Capt. A. W. Clarke** (Trinity House) announced there was an overflow meeting downstairs of some 300 people waiting to hear Sir Rosslyn Wemyss, and he wished to tell him before he went downstairs to it, as the First Sea Lord of the Admiralty, that the Devonport Fund had now reached the sum of £115,000. The Lords Commissioners of the Admiralty and the generous donors might rest assured that it would be well and economically spent, and the new buildings of the Dreadnought Hospital would be in conformity with that beautiful building of Wren's in close proximity. The Lords of the Admiralty had granted a generous lease, and were helping the hospital in every way in their power. He welcomed that magnificent meeting, that representative and influential audience before him and the overflow meeting downstairs, as tributes in themselves to the merchant seaman, showing how deep and abiding was the interest taken in him by the general public. Speaking as a merchant seaman, he declared that Providence helps those who help themselves. We are going to help ourselves. We are going to make good those losses caused by U-boats in due time, because ships are the life of the Empire, but there is one thing that no one can improvise, and that is the spirit that makes the men go down to the sea in those ships and face the devil and all his works upon the ocean. That we are content to leave to the men themselves, being fully assured that that spirit is and will be forthcoming, and that the men are and will always be ready. It was for the public and for those present to give encouragement to these splendid men by assuring them that in their time of trouble and distress, in sickness and sorrow and misery, they would have hospitals and other good things ready to receive them on shore. Was there anything too much that the landsmen and women could do for the men on the ships of the Merchant Service which come and go despite the war, although it was peculiarly concentrated upon their destruction? Yachtsmen, who thought it a pleasure to go to sea before, are now patrolling the coasts day and night, and doing it well. The fishermen are in mine-sweepers sweeping the commercial channels all round our shores. That is what these brave men are doing, despite the devil and all his works at sea. It has been borne in upon all the sailors of the Royal Navy, the Merchant Service, yachtsmen, or fishermen, that they are all members of the same united brotherhood. The war has drawn closer together the links which bind the great Dominions beyond the seas with this island. Captain Clarke saw the men of the *Sagamore* when they came to England, and afterwards some of them in the Dreadnought Hospital. All their limbs were amputated, some above the knee and some below, and it was most pathetic to see the joy of the men who had saved their knees so that they could make use of artificial limbs. What was done for those men? South Africa subscribed a large sum of money, and these men were fitted with limbs at no cost to themselves, whilst each man had a sufficient sum to set him up in the business that he was best able to carry on. That is what the Empire was doing. The other day something like £20,000 was subscribed by Chinese, Indians, Cingalese, Tamils, and others for the sailors, and their message expressed their loyal and humble duty to His Majesty the King, and rejoiced that Lieutenant H.R.H. Prince Albert had been appointed to the *Malaya*, the ship they sent over for the Empire. Capt. Clarke, in conclusion, hoped that the meeting, and many outside, would remember, as the little child said at grace, **"to thank God and the sailors for every mouthful I eat,"** that they would recognise the work of the seamen, and that they would give the Dreadnought further support for what the seamen had done and were doing for everybody.

**Admiral Sir Henry Jackson** urged the establishment of a Convalescent Home for the Seamen's Hospital. The meeting then terminated.

